# Microcarcinoid and Ulcerative Colitis: Case Report and Literature Review

**DOI:** 10.7759/cureus.8803

**Published:** 2020-06-24

**Authors:** Denzil Etienne, Andrew Ofosu, Mel A Ona, Madhavi Reddy

**Affiliations:** 1 Gastroenterology and Hepatology, The Brooklyn Hospital Center, Academic Affiliate of the Icahn School of Medicine at Mount Sinai, Clinical Affiliate of the Mount Sinai Hospital, Brooklyn, USA; 2 Gastroenterology, The Brooklyn Hospital Center, Affiliate of the Mount Sinai Hospital, Brooklyn, USA; 3 Gastroenterology and Hepatology, Pali Momi Medical Center, Honolulu, USA; 4 Gastroenterology and Hepatology, The Brooklyn Hospital Center, Affiliate of the Icahn School of Medicine at Mount Sinai, Brooklyn, USA

**Keywords:** inflammatory bowel disease, microcarcinoid tumor, rectal neuroendocrine tumor

## Abstract

Gastrointestinal microcarcinoid tumors are rare, and the concomitant diagnosis of microcarcinoid tumor and inflammatory bowel disease is even rarer. A 54-year-old African American male with an eight-year history of ulcerative colitis (UC) presented with a three-day history of abdominal pain and bloody diarrhea. Rectal biopsy on colonoscopy was notable for small nests of neuroendocrine cell proliferation in the mucosa consistent with a diagnosis of microcarcinoid tumor. Whether the incidence is coincidental or represents an epiphenomenon of chronic inflammation remains to be determined.

## Introduction

Inflammatory bowel disease (IBD) has been shown to promote the development of colorectal adenocarcinoma, especially when the duration of disease has been prolonged [1,2]. Interestingly, colonic carcinoid and microcarcinoid tumors have also been commonly reported amidst the backdrop of IBD, suggesting an association that may be more than coincidental. We present one case of rectal microcarcinoid in the setting of ulcerative colitis (UC).

This case was previously presented at American College of Gastroenterology (ACG) conference on October 22, 2017 [3].

## Case presentation

**Figure 1:**
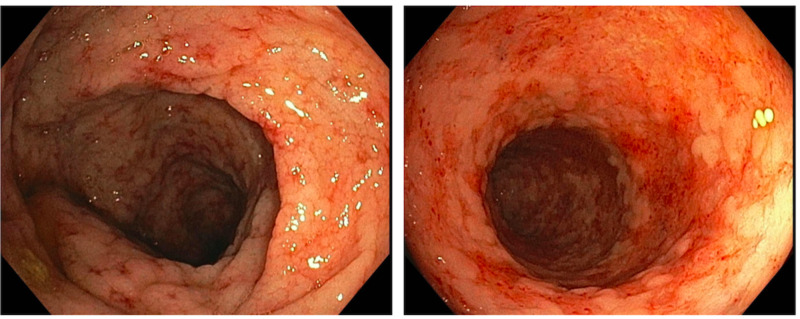
Severely erythematous mucosa.

A 54-year-old African American male with an eight-year history of UC, systemic lupus erythematosus, and medication non-compliance presented with a three-day history of abdominal pain and bloody diarrhea. Physical examination was unremarkable. Colonoscopy was performed and demonstrated edematous, friable, and severely erythematous mucosa extending from the rectum to the cecum consistent with pan-ulcerative colitis as shown in (Figure 1) [3].

**Figure 2:**
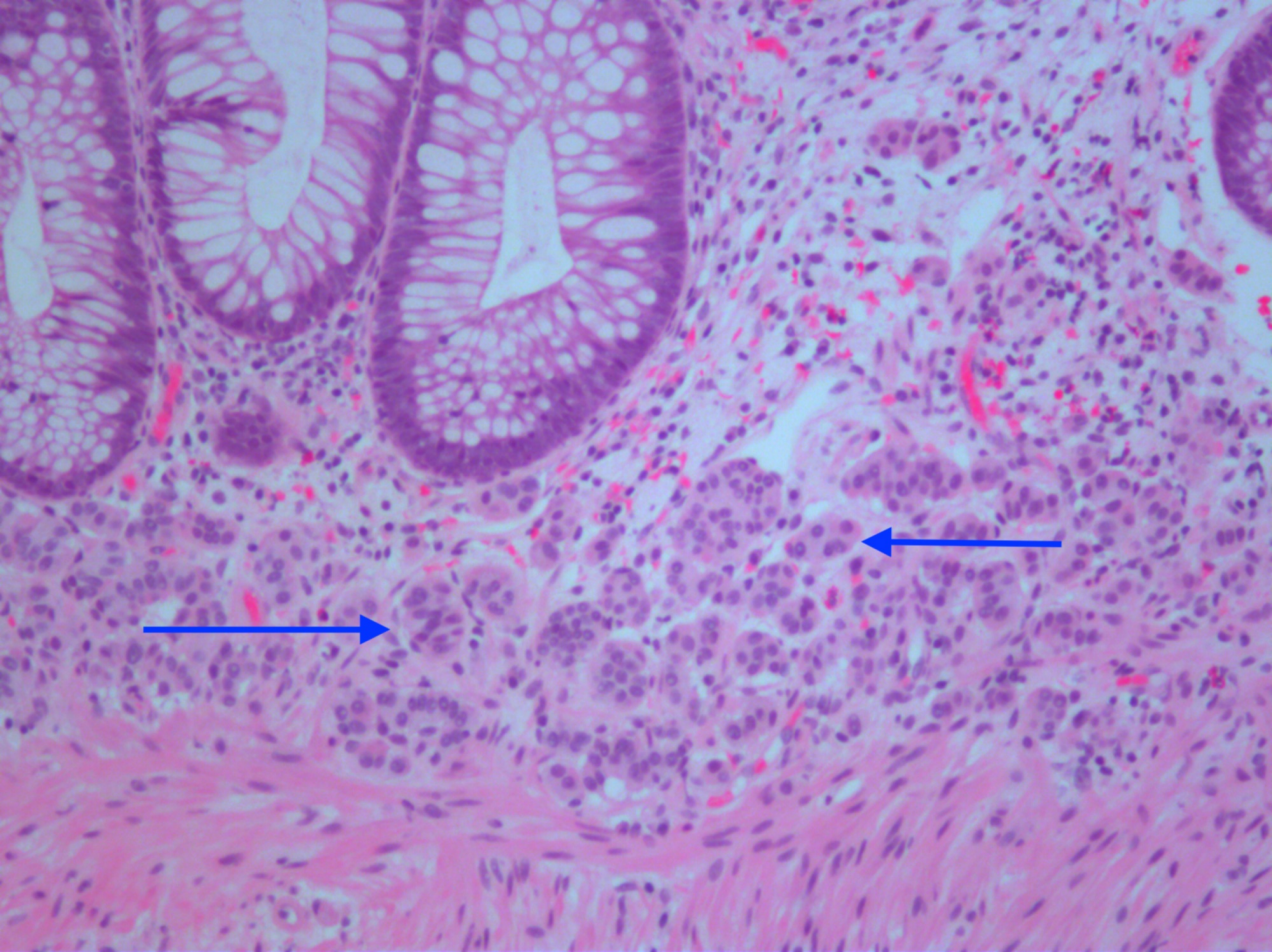
Small nests of neuroendocrine cell proliferation in the mucosa

Random biopsies were taken approximately every 10 cm from the cecum to the rectum with subsequent histopathology confirming moderately-active chronic colitis in every segment. The rectal biopsy was also notable for small nests of neuroendocrine cell proliferation in the mucosa, consistent with neuroendocrine tumor (NET) (Figure 2) [3].

The mucosal microcarcinoid tumor nests were confirmed by positive staining for CD56 and synaptophysin. Staining was negative for chromogranin. There was no ulceration or evidence of granulomas. He was subsequently discharged on a tapering dose of oral steroids, mesalamine (oral and rectal enemas), to follow-up at the gastroenterology clinic. He was lost to outpatient follow-up but was re-admitted to the hospital three weeks later with similar symptoms. He reported non-compliance to medications since the time of discharge and was started on intravenous steroids and mesalamine (oral and rectal enemas). The patient showed significant improvement in symptoms, and flexible sigmoidoscopy/rectal endoscopic ultrasound (EUS) was performed demonstrating healed mucosa. Rectal biopsies showed inactive chronic proctitis with no evidence of mucosal/submucosal neuroendocrine cells. CT scan of the abdomen and pelvis was unremarkable, and octreotide scan showed minimal patchy uptake in the rectum with no evidence of remote metastasis. He was discharged on a tapering dose of oral steroids, mesalamine (oral and rectal enemas), to follow outpatient.

## Discussion

UC has been shown to promote the development of colorectal neoplasms (including adenocarcinoma and less commonly malignant lymphoma), especially when the duration of the disease has been prolonged [1,2].

Interestingly, colonic carcinoid and rectal NETs or microcarcinoid tumors have also been commonly reported amidst the backdrop of UC, suggesting an association that may be more than coincidental. With regard to UC, various authors have suggested that local carcinoid tumors may be more likely to arise when there is enteroendocrine cell hyperplasia, as can be seen in states of chronic mucosal irritation/injury such as bronchiectasis and type A chronic gastritis [4,5].

Enteroendocrine cells comprise a heterogeneous population of cells (typically located at the base of the intestinal crypts or in the adjacent lamina propria), thought to be of endodermal origin, dispersed throughout the gastrointestinal tract as part of the diffuse endocrine system, previously termed the amine precursor update and decarboxylation (APUD) system. These specialized cells produce a variety of different polypeptide/trophic hormones responsible for various regulatory activities within the gastrointestinal system and show varying degrees of affinity for silver salts (hence the terminology of argentaffin/enterochromaffin cells and argyrophilic cells used in earlier literature). Furthermore, it has also been suggested that levels of trophic hormones may increase when enteroendocrine cell hyperplasia occurs secondary to chronic mucosal irritation/injury, possibly promoting malignancy (adenocarcinoma, lymphoma, or carcinoid) in IBD [6].

Two theories have been posited regarding the development of carcinoid tumors. The first involves the emergence of these tumors as a consequence of the neoplastic transformation of endocrine cells. Another theory holds that these tumors are derived from primitive pluripotent endodermal cells [7].

Rectal carcinoids account for only 9% of gastrointestinal carcinoids and 1% of all rectal malignancies [8]. They can be classified into three types: “true” carcinoid or argentaffinoma, “atypical” or non-argentaffin carcinoid, and carcinoid showing mucin positivity. The most prevalent type, the atypical or non-argentaffin carcinoid, is generally benign [8].

Rectal NETs (or microcarcinoids) represent small nests of gut endocrine cells that exist as mucosal/submucosal solitary nodules or solid endocrine growths larger than 500 µm but which have not formed macroscopic tumors. They are usually found in inflamed areas and are less than 1 cm in half of the cases. Rectal microcarcinoids are generally asymptomatic, with a five-year survival of 72%-89% and only 5% of cases progressing to carcinoid syndrome [8]. The actual incidence and prevalence of rectal microcarcinoids in UC remain unclear.

As noted earlier, enteroendocrine cells have been shown to undergo proliferation in response to mucosal injury, which has been postulated to induce neoplastic (including microcarcinoids) development [6]. Interestingly, complete regression of microcarcinoids that were found in diseased segments of the intestinal tract has also been reported when the inflammatory conditions (e.g. chronic gastritis, UC) were treated [9]. However, these findings should not be interpreted in isolation, as an understanding of the natural history of proliferative/regressive changes in gut enteroendocrine cells remains imperfect. In fact, cases exist demonstrating the presence of carcinoid/microcarcinoid tumor in UC patients with no associated proliferative changes in gut enteroendocrine cells [10]. Other authors have reported conflicting results regarding the associated proliferative changes in enteroendocrine cell population in patients with UC. Using the Fontana stain, Skinner et al. reported a decrease in enteroendocrine cell burden in colonic specimens of patients with UC [11]. The decrease in enteroendocrine cells did not correspond to the severity of colitis. Verity et al. used the diazo reaction to study portions of resected colons from patients with UC and also noted a decrease in argentaffin cell population that correlated to the severity of mucosal inflammation [12]. Using similar techniques, Peterson and Watson found an increase in the number of enteroendocrine cells using the diazo reaction [13]. Microcarcinoids have also been sporadically found in segments of colon unaffected by inflammatory conditions [14].

Thus, the association between carcinoid tumors and UC remains unclear given a similar incidence of carcinoid tumors in patients with and without IBD as well as the tendency to find the tumors in portions of bowel typically unaffected by UC (e.g. appendix and ileum) [15]. The presence of microcarcinoid in the appendix does not, however, preclude a correlation between the two conditions as subsequent investigations have confirmed UC involvement of the appendix [16].

## Conclusions

Our case described the disappearance of mucosal neuroendocrine cells in the rectum of a UC patient who had clinical and histopathologic resolution of active disease following treatment. Despite the absence of neuroendocrine cell hyperplasia in this patient, the findings appear to support a link between chronic inflammation and the development of microcarcinoids. This potential correlation warrants further investigation as the precise role that chronic inflammation plays on the development of microcarcinoids remains unknown. A logical corollary would also involve exploring the utility of microcarcinoids as a surrogate marker for the severity of disease in UC patients. 
